# Stakeholder engagement vs. social distancing—how does the Covid-19 pandemic affect participatory research in EU marine science projects?

**DOI:** 10.1007/s40152-021-00223-4

**Published:** 2021-05-14

**Authors:** Vera Köpsel, Gabriel de Moura Kiipper, Myron A. Peck

**Affiliations:** 1 Institute for Marine Ecosystem and Fishery Science (IMF), University of Hamburg, Große Elbstraße 133, 22767 Hamburg, Germany; 2 Department of Coastal Systems (COS), Royal Netherlands Institute for Sea Research (NIOZ), PO Box 59, NL-1790 Den Burg (Texel), AB Netherlands

**Keywords:** Stakeholder engagement, Covid-19, Social distancing, Marine research, Transdisciplinarity, EU

## Abstract

In the realm of marine science, engaging with stakeholders (e.g., industry members, policy-makers, managers, NGOs) is an important method applied in many research projects. The Covid-19 pandemic has severely impacted this engagement in two ways. First, social distancing measures forbid most face-to-face participatory activities originally envisioned in projects. Second, the restrictions have caused hardships for the stakeholders being engaged by these projects. We assessed the impact of Covid-19 on stakeholder engagement activities in ongoing EU marine science projects by posing the following questions: What problems has Covid-19 caused for the workflow and outcomes of current research projects, (ii) how have scientists responsible for stakeholder engagement coped with the situation, and (iii) if alternative stakeholder activities were implemented, how have these been evaluated? Our survey was conducted nearly 2 months after the onset of Covid-19 lockdowns. It addressed researchers who engage with stakeholders in EU projects and delivers insights into the practical implications of Covid-19 for stakeholder engagement and the measures taken to tackle this challenge. The paper highlights the impacts of the pandemic on stakeholder engagement in marine science, outlines current coping strategies in different EU projects, and recommends seven practical actions to promote and maintain meaningful exchange with stakeholders in times of social distancing and lockdowns.

## Introduction

Engagement with stakeholders has become a key method in environmental research projects over the past decades (Brinkmann et al. 2015; Chilvers and Kearnes 2016). This development in marine science mirrors a wider turn towards participatory research over the past 20 years and has led to the opening up of a traditionally natural science-dominated research field towards practice actors (Rauschmayer et al. 2009; Linke and Jentoft 2016; Linke et al. 2020). The involvement of non-academic actors allows the inclusion of diverse viewpoints, increases the trust in and acceptance of scientific results, and “promot[es] social learning where stakeholders learn from each other and build new knowledge while developing new relationships” (Sterling et al. 2017: 160). Funding agencies such as the European Commission (EC) have fostered this development by including stakeholder engagement as an expected impact of funded projects (Scherer et al. 2018), and this participatory approach is embedded in many, ongoing projects (see e.g. PANDORA Project 2020; Black Sea CONNECT 2020; Linke et al. 2020).

We have written this paper not only as researchers working in marine natural and social science but also as members of H2020 marine projects tasked with the coordination of stakeholder engagement (SE) across Europe. In this capacity, we have witnessed first-hand the strong effect of Covid-19 on engagement in several ways. First, the measures taken by governments to slow down the spread of the virus have changed general work practices in academia (see the “Academic life in a new, digital mode” section). Second, social distancing measures make most of the hands-on participatory activities originally planned impracticable (see the “Social distancing and SE in EU projects” section). Third, hardship (economic or otherwise) caused by the pandemic has likely reduced the priority of stakeholders to commit time in non-essential activities such as participatory events (see the “Covid-19 impacts on fishing and the marine sector” section). The impact of the Covid-19 pandemic is historically unparalleled even in comparison with other large-scale incidents such as natural disasters. While such sudden events come with “structural damages to buildings, […] losses of data, reagents, research animals, unique equipment and materials” (Chinen 2018: 876), Covid-19 has had very different impacts on the academic world by restructuring work routines and causing isolation (see the “Academic life in a new, digital mode” section).

The central objective of this paper was to explore how the Covid-19 pandemic has impacted SE activities in European marine science projects. We conducted an online survey among EU H2020 projects in the fields of marine biology, ecology, modelling, and technology which cooperate with stakeholders. The online survey was conducted between June and July 2020, nearly 2 months after the onset of Covid-19 lockdowns, and was a cooperation between the EU projects PANDORA (fisheries science) and SENTINEL (renewable energy science). Our survey attempted to address three sub-questions:

1. What problems has Covid-19 caused for the workflow and outcomes of current research projects so far?
2. How have the scientists responsible for organising engagement activities coped with the situation?
3. If alternative stakeholder activities were implemented, how were they evaluated?

We complement our survey results with a review of the existing literature on the impacts of Covid-19 on science and academia per se, on EU research projects and their stakeholder engagement, and on the lives of the stakeholders engaged. Our hope was to deliver insights into the practical implications of Covid-19 for SE and the measures taken by projects to tackle this challenge. This paper highlights the impacts of the pandemic on participatory research, outlines coping strategies of ongoing EU projects, and gives practical recommendations on how to maintain meaningful exchange with stakeholders in times of social distancing and lockdowns.

## Stakeholder engagement in the marine sciences

Marine science was traditionally conducted by researchers in the natural sciences such as biologists, oceanographers, and ecologists, and the consideration of human activities in marine and coastal policy and management “has historically been lacking” (McKinley et al. 2020: 85). Over the past two decades, however, there has been a realisation that sustainable management and use of marine resources cannot only take a natural science perspective; it is crucial to include research on the societal contexts of management and decision-making (Bavinck and Verrips 2020).

Mackinson et al. (2011) stressed that effective management of the seas is only possible through cooperation with stakeholders to incorporate their perspectives and experiential knowledge so that the questions, design, and outcomes of research projects are meaningful to society. The engagement of stakeholders is a central tool of transdisciplinarity, a research approach that “aspires to make the change from research for society to research with society” (Aps et al. 2020: 214, referring to; Scholz 2000) and helps integrate different knowledge systems of scientists and practice actors such as policy-makers, industry members, and/or conservationists (Hirsch-Hadorn et al. 2008; Aps et al. 2020).

A transdisciplinary approach is central for investigating topics such as natural resource management or the so-called tragedy of the commons (Brinkmann et al. 2015). Sterling et al. (2017: 160) highlighted that “since natural systems are connected to so many social-cultural domains, it is important for SE efforts to consider the social dimensions” of any environmental management process. To meet this demand, more and more research projects have implemented active exchange with non-academic actors (see Peck et al. 2020; ClimeFish 2020; Mackinson and Holm 2020). Levels of engagement range from very low (e.g. disseminating results and information), medium (e.g. consulting stakeholders during the research process), high (e.g. cooperation or collaboration in obtaining research results), to very high (co-leading projects with stakeholders) (see Stauffacher et al. 2008 for details). This development highlights the importance of cooperating with practitioners to produce sound science and solve local problems (e.g. Mackinson et al. 2015; CIESM 2018). Mirroring the increased importance of SE, the body of literature on transdisciplinarity (e.g. Richler 2020; Runde 2019; Silberberg and Martinez-Bianchi 2019) as well as the repertoire of best practice guidelines (e.g. Open Channels 2020; UNESCO 2020) has continuously grown. The consensus is that including stakeholders makes research results more societally relevant, more robust, and more easily understood and accepted outside academia.

### A growing interest in exchange with practitioners

Although the term “stakeholder” is used differently across disciplines and the usefulness of the term is debated (e.g. Miles 2012; Griffin 2017; Colvin et al. 2020), in this study, we refer to “stakeholders” based on the definition by Freeman (1984) as any persons and groups who can directly or indirectly affect a decision or endeavour, or are affected by it (see also Sterling et al. 2017). The stakeholders engaged in marine science projects cover a diverse array of societal groups from fishers and aquaculture farmers and their representatives, to related industries, coastal human communities, agencies responsible for marine management, and non-governmental organisations (NGOs). Depending on the nature of a research project, scientists external to a project can also be viewed as stakeholders (Mackinson et al. 2011).

In fisheries science, the reformed Common Fisheries Policy (CFP) of 2002 constitutes an important milestone on the road towards engaging with actors from outside of academia. The ambitious aims of this policy have paved the way for increased involvement of stakeholders in fisheries governance and research. Increasing the common ground between scientists and practitioners was viewed as an important prerequisite for implementing the CFP (Mackinson and Holm 2020). The Europe-wide GAP and GAP2 projects explored how SE can be conducted most efficiently for both fisheries scientists and stakeholders (Mackinson et al. 2015; Holm et al. 2020). Following the epistemological shift showcased by the GAP projects, more and more projects in the natural sciences have taken up SE as part of their methodology (Steins et al. 2019; Mackinson and Holm 2020). Moreover, a relatively new field of marine social science has emerged that focuses on the relationships between people and the sea, the values ascribed to coasts and oceans, and related human activities (for a detailed introduction to marine social science, see McKinley et al. 2020). Marine social science “include[s] a diverse […] set of disciplines, methods, and theories that can be applied to rigorously study the human dimensions of ocean and coastal issues and challenges” (Bennett 2019: 247). Engagement and exchange with stakeholders from the maritime sector is one of such methods, and it is well-established in marine social science. Although there is a long track record of research examining human decision-making in marine management and the perspectives of practitioners on marine environments and resources (e.g. Dale and Armitage 2011; Coll et al. 2014; Stange et al. 2015; Stephenson et al. 2017), incorporating societal values and viewpoints into natural scientific marine research is a newer development (Cvitanovic et al. 2015; Mackinson and Holm 2020).

When engagement works well, it can significantly improve the relationships between scientists and members of the fishing and aquaculture sectors by building mutual trust and understanding for each other’s viewpoints (Hartley and Robertson 2006). Nonetheless, engaging practitioners in research projects remains a challenge. Engagement is shaped by which stakeholders are identified and invited to participate (Metzger et al. 2017; Linke and Jentoft 2016). Cvitanovic et al. (2015: 39) argue that “cultural differences” between scientists and stakeholders (e.g. differing value systems) as well as differences between different stakeholder groups can lead to misunderstandings. Additional factors hindering successful engagement are the absence of methods training, limitations set by research institutes, and a lack of knowledge of the information needs of stakeholders (Cvitanovic et al. 2015). Moreover, it can be challenging to integrate stakeholder knowledge, which is often qualitative and experiential in nature, with qualitative scientific data that tends to be viewed as more objective and systematic (Stange 2017; Köpsel 2019). Engagement, therefore, requires much dedication, time and energy from both scientists and stakeholders (Brinkmann et al. 2015).

### Increased importance of  stakeholder engagement  in research funding

The importance of engagement with stakeholders has been recognised not only in the academic world but also by research funders globally. As one of the largest research funding bodies worldwide, the EC recognised the importance of SE by applying a Responsible Research and Innovation (RRI) paradigm as part of its “Horizon2020” scheme. According to the EC (2020), the RRI paradigm “implies that societal actors […] work together during the whole research and innovation process in order to better align […] with the values, needs and expectations of society”. The EC, thus, clearly takes the standpoint that “active exchange between research and stakeholders is the prerequisite for the successful uptake of research results in policies” (Gramberger et al. 2015: 202). Similar trends can be observed across the globe, for instance in the USA and Australia, where research funding bodies put increased emphasis on engagement with society (The National Science Foundation 2020; Australian Research Council 2020). In this context, Mackinson et al. (2011: 20) underscore that scientists and stakeholders should cooperate closely by jointly “identifying, prioritizing, planning, doing, interpreting, evaluation and communication of the research” during all stages of research projects.

Considering the new requirements by funding bodies, the majority of EU H2020 projects embrace some level of SE, even in projects based on research topics that did not traditionally apply a transdisciplinary approach (EC 2017; Mackinson and Holm 2020). Although the marine social sciences have considerable experience in the engagement of stakeholders (cf. “A growing interest in exchange with practitioners”), funding requirements have led to a substantial increase in SE within projects composed of mainly natural scientists who lack training in exchanging with stakeholders (Peck et al. 2020). Covid-19 restrictions pose an additional burden on such projects because trained personnel are important to successfully modify activities to maintain stakeholder engagement. Furthermore, large EU projects submit a stakeholder engagement strategy that outlines key stakeholders, goals, formats, and timelines of SE (see e.g. PANDORA Project 2018). Large deviations from this strategy require explanation and could have negative consequences to the success of research project (EC 2020c). In most cases, the emergence of Covid-19 has demanded that engagement strategies be re-defined.

## Research(ers) and stakeholders in “Covid mode”

### Academic life in a new, digital mode

Most countries in the world issued distancing and lockdown measures in spring 2020 which, at the time of writing (August 2020) and revising (November 2020 through March 2021) this article, have been lifted and re-sanctioned to varying degrees. (Self-)isolation, home office, and a halt of leisure activities burden everyone, both project scientists or stakeholders alike (Brooks et al. 2020). Work routines have been transformed, and uncertainty remains on the horizon regarding the long-term consequences on work, education, social relations, the economy, and the environment (Corbera et al. 2020: 191). For many in academia, this is a time of online meetings, re-shaping teaching methods, and learning to cope with working from home often with unforeseen family care duties (Corlett et al. 2020). Where laboratory work must continue, time-consuming protocols and distancing measures have been put into place (Gewin 2020). During this period, many science conferences and symposia have either been cancelled or moved to the digital sphere (e.g. ICES 2020; ICYMARE 2020). Considering this range of changes, our survey sought to understand how the lives of academics have been affected by the pandemic, how the pandemic has impacted their work in research projects, and how academics perceive the pandemic has impacted their project’s stakeholders.

#### Two sides of a (digital) coin

Beaunoyer et al. (2020) highlighted that, in times of the pandemic, digital technologies are “one of the only remaining vectors for […] social interactions to take place”. The shift towards digital tools in academic research enables us to maintain communication despite social distancing measures. Virtual tools offer great flexibility for participants and enable stakeholders to partake in project meetings from home (Davis et al. 2019). They provide the opportunity of participation across large geographical distances and overcoming travel time and costs, thus being much more inclusive than physical events. Klöwer et al. (2020) reported that the number of attendees rose from 16,000 pre-Covid to 26,000 at the annual meeting of the European Geosciences Union (EGU) due to digital participation in 2020. This increased participation occurs alongside a 90% reduction in the CO_2_ emissions caused by the event (Klöwer et al. 2020). Virtual engagement, therefore, bears enormous advantages for relieving pressure on the environment. The extensive use of digital tools, however, has several disadvantages. Back-to-back online meetings leave little time for numerous other tasks. Beaunoyer et al. (2020) argued that, when digital tools become the key form of communication, certain societal groups are inevitably excluded from the conversation. Whether it is the elderly, students with little money for good laptops or groups without access to internet facilities, the more digital we go, the more people we leave behind. It was important, therefore, to ask survey participants whether there were stakeholder groups they could not reach via online tools during the pandemic.

The pandemic has also impacted research methods and strategies, and it is important to question the validity of data collected during this period. In this context, Fell et al. (2020: 1) stressed the importance of ensuring that “conclusions drawn from data collected during the pandemic are valid, representative, [and] generalisable to a post-pandemic world, and comparable to a pre-pandemic one”. Fell et al. (2020) provided a list of recommendations to help assess the validity of research results including whether (1) additional variables were collected in the gathered data (e.g. data on research location can help identify bias caused by the pandemic), (2) social distancing measures were considered in the study design and choice of methods, and (3) results were interpreted with respect to points 1 and 2. These generic recommendations also apply to SE. Our survey, therefore, included a question on how scientists ensured the validity of data collected to assess how the circumstances caused by Covid-19 are considered in current engagement practice.

### Social distancing and stakeholder engagement in EU projects

It is the very core of this article to explore how SE activities in marine science projects are affected by the Covid-19 pandemic. At this point, peer-reviewed literature examining the impacts of social distancing on transdisciplinary research is still scarce; nonetheless, we would like to provide a brief background on how research in EU projects has been impacted by the pandemic. As researchers with many years of experience in EU H2020 projects, we can report that lockdowns have significantly changed our work. Travel, workshops, and face-to-face exchange with colleagues from other countries are integral parts of project life. It is how relationships within and beyond the project are kept alive, and results are compared and integrated. To understand the impacts of the pandemic on different formats of SE, we inquired from survey participants which methods of engagement were foreseen in their projects pre-Covid and which alternatives were chosen to adapt to social distancing.

The fact that Covid-19 may have strong effects on the progress of research projects was recognised by the EC early into the pandemic. When travel restrictions were issued in March 2020, the EC declared the pandemic a “force majeure” case. Therefore, planned travels that could not be carried out could be reimbursed from project budgets (for “force majeure” regulations, see EC 2018). Moreover, a FAQ website was set up by the EC which addresses frequently asked questions regarding delays in the workflow of projects or payment of salaries for project staff who cannot currently work. Among others, it is stated that an extension of the project duration of up to 6 months is possible due to delays caused by Covid-19 circumstances (ibid). These measures relieve project budgets and clarify what happens in the case of delays; what they did not provide, however, was a solution for the fact stakeholder engagement activities that relied on face-to-face meetings could no longer be conducted. Aware that the success of projects may depend on when stakeholder workshops are held and data is collected, we included questions about the overall project goals and workflow in our survey.

The EC did not provide any guidelines for engaging stakeholders in projects, neither do they offer solutions for how to handle engagement in times of Covid-19. One of the few existing guidelines addressing Covid-19 are those published by OXFAM, who advise their staff to consider several important points when engaging with stakeholders during the pandemic. Among others, they recommend to assess the information needs of different stakeholder groups as well as their preferred communication channels; consider how to reach out to stakeholders who do not have access to digital technologies; and make use of the entire array of remote communication technologies, from video calls to radio and telephone (OXFAM 2020). Focusing on research projects and the marine sector specifically, we provide a second set of recommendations at the end of this paper to support scientists in keeping SE alive in times of the pandemic.

### Covid-19 impacts on fishing and the marine sector

The social distancing measures enacted by governments across Europe since March 2020 affected all sectors of the economy. The contributions to this special issue illustrate the widespread impacts of the Covid-19 pandemic on all societal groups involved in fishing, fisheries management, and the use and management of marine resources. With peer reviews still in process, much of what we can learn about the impacts of the pandemic on the marine sector is still not published. Early results from Venetia (Italy) from March/April 2020 showed that, even at the start of lockdowns, vessel and fishing activities were already reduced by 69% and 84%, respectively, in comparison to the same months in 2017 (Depellegrin et al. 2020). Food prices for fish and seafood increased significantly especially in countries with strict lockdown measures between January and March 2020, manifesting an already decreasing consumer demand (Akter 2020). The Food and Agriculture Organization (FAO) (2020) reported severe negative impacts on the fishing and aquaculture sector not only due to closures of supermarkets, hotels, and restaurants but also by difficulties in market access and closed national borders. The EC (2020a) clearly stated that fishing and aquaculture are among the sectors suffering the largest economic losses due to the pandemic. These developments are challenging the entire sector but pose particular burdens on small and family-owned businesses whose livelihood solely relies on income from marine resources (Kraus et al. 2020). With fisheries and aquaculture crucial for global food security, the lockdowns and resulting restrictions in economic activity highlight the complex connections between local and global markets, supply and demand, and the livelihoods of coastal communities (Knight et al. 2020).

From a conservation perspective, on the other hand, it is arguable that the reduction in (air) travel and in some other economic activities may offer an opportunity for the environment to recover from human-induced pressures (Pearson et al. 2020). During the lockdown, the levels of pollutants in rivers and seas have declined (Pinder et al. 2020). Fishing pressure on European stocks has fallen to pre-World War II levels (Kemp et al. 2020). Although the Covid-19 pandemic potentially contributes to the recovery of living marine resources, however, both scientists and practitioners in the marine sector have faced negative, short-term effects.

## Study design, survey development, and data analysis

The survey underlying this paper was as a cooperation between the EU H2020 projects PANDORA and SENTINEL. The idea was born in March 2020, when researchers from both projects responsible for SE discussed the difficulties brought about by social distancing. The central method of this study was an explorative, semi-quantitative, self-completion questionnaire (cf. Bryman 2012: 186). It is semi-quantitative due to the use of different question formats, from Likert-like scales (ibid: 239) to multiple-choice questions and free text boxes. Free text boxes were used where the responses to multiple-choice questions should be explained in more detail. The survey was carried out with the online tool “LimeSurvey” (LimeSurvey 2020). The link to the questionnaire was distributed via email to the coordinators of all current EU H2020 marine science projects by the EC’s Policy Officer for Healthy Seas and Oceans. Our sample size of 30 projects, therefore, equals all ongoing H2020 marine science projects. In addition, we advertised the survey via Twitter and ResearchGate to reach fellow scientists. As previously stated, the stakeholders of these projects were not surveyed. We clearly specified that the survey should be completed by project members responsible for planning and carrying out SE. The analysis included two steps. First, quantitative survey responses were compiled and compared (cf. Creswell 2018: 155ff). Second, the results were complemented by and interpreted on the basis of the additional information from the free text replies.

### Limitations of this study

Due to its explorative nature, our survey has some limitations. In critical reflection, we would like to raise a few points regarding its generalisability, “project bias”, and the timing of this study, among others.

#### Generalisability

One might argue that the number of completed surveys is not sufficient for a quantitative analysis, and from a purely statistical viewpoint, this is correct (Bryman 2012: 176). However, our aim was not to reach generalisability in our results, but to gain an overview of the current status of marine science projects. As only 30 EU H2020 marine projects were ongoing, 24 responses covering 12 EU projects plus five other projects provided us with a good first insight into how SE was affected by pandemic restrictions.

#### “Project bias”

Bryman (2012: 187) defined bias in surveys as “a distortion in the representativeness of the sample”. In our case, the survey was completed by differing numbers of respondents from different projects, which constitutes a certain “project bias”. In questions regarding workflow, multiple replies from one project may bias results. Regarding methods, alternatives, and their evaluation, however, this bias does not apply as different scientists from one project use different approaches for engaging stakeholders.

#### Timing of the survey

Our survey was carried out fairly early in the pandemic between June and July 2020. As Covid-19 and the response of governments have changed rapidly, the results presented here show only an initial snapshot of impacts on SE as of summer 2020. It is, therefore, advisable to repeat the survey at a later time to assess the final influence of the restrictions on each project.

#### No first-hand stakeholder perspectives

Our findings of Covid-19 impacts on the lives of stakeholders were based on existing literature and the perceptions of the project scientists. Although the impacts raised by the literature and the survey correspond well, it is important to state that we did not collect first-hand stakeholder perspectives.

Acknowledging these limitations, we hoped to provide valuable, initial insights into the impacts of Covid-19 on SE activities in Europe-wide marine science projects and illustrate alternative strategies applied by different projects.

## Covid-19 impacts on (EU) marine science projects

### Overview of surveyed projects, motivations, and methods of engagement

**Fig. 1:**
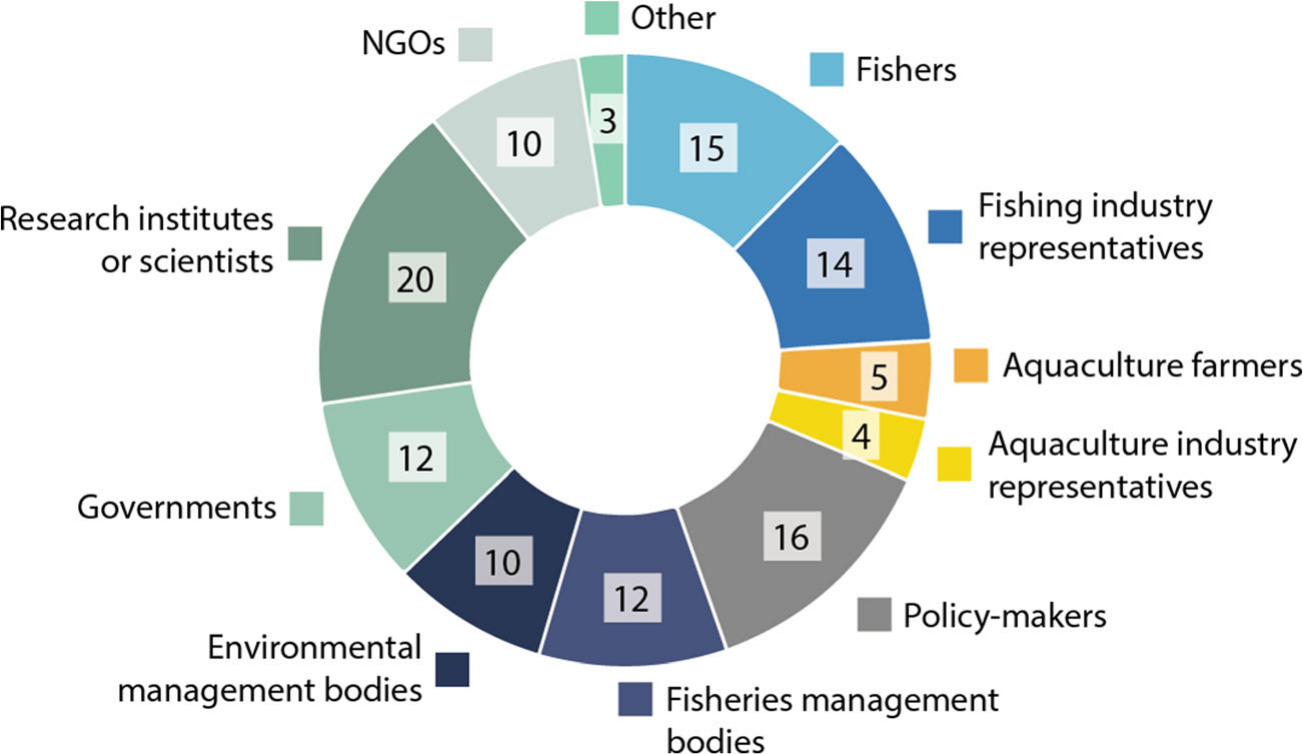
Stakeholders groups engaged in current European marine science projects (multiple responses possible, *n* = 24). Source: survey data

**Fig. 2:**
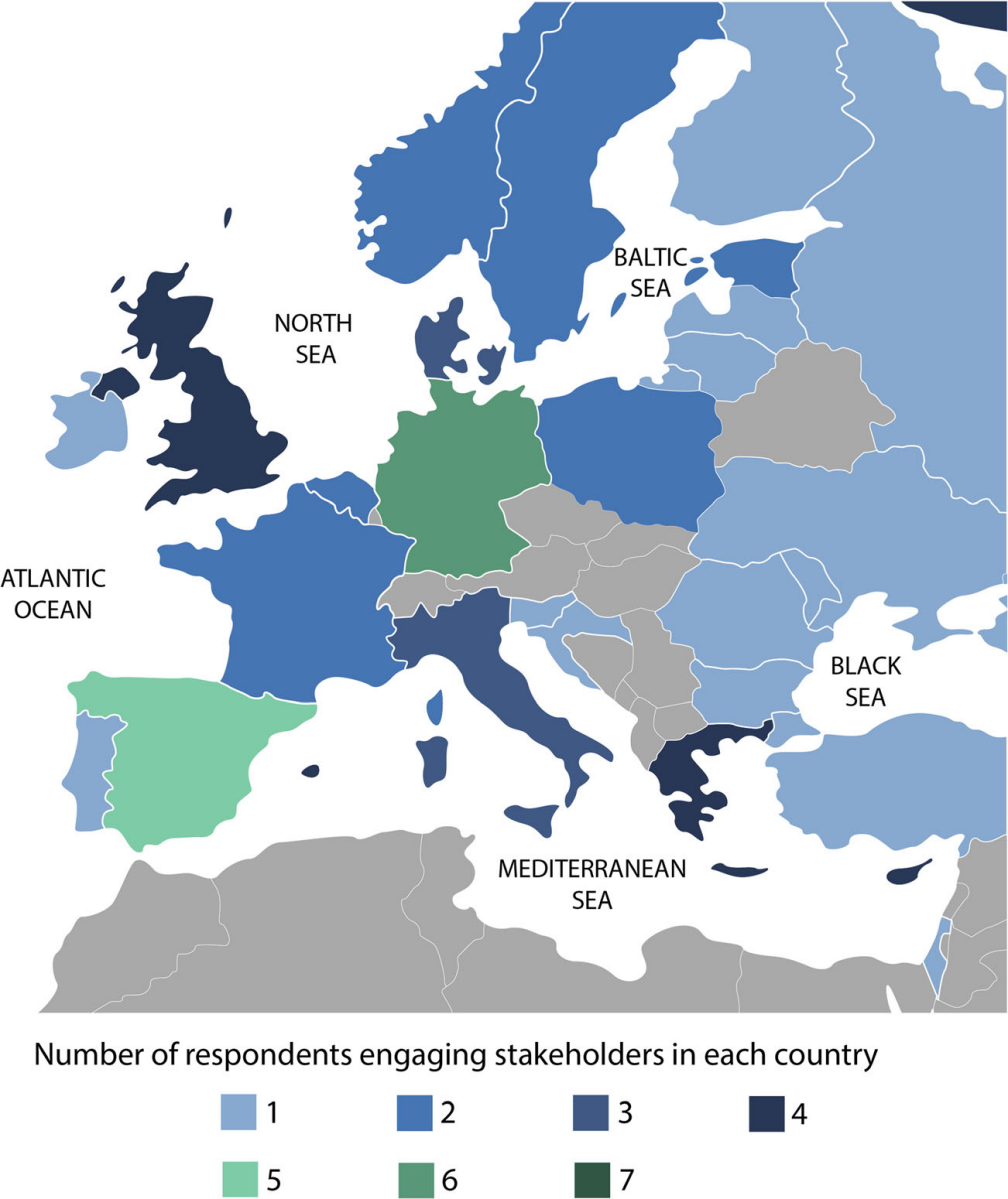
Countries in which the surveyed projects engage with stakeholders. Source: own figure based on survey data

In total, 24 surveys were completed from twelve EU H2020 marine science projects. This constitutes a return rate of 40% for the survey invitations that were sent directly to project coordinators by the EC. The number of replies per project range from one to seven, reflecting differences in the number of project members responsible for engaging stakeholders. In addition, scientists from three projects funded by national institutions, one BONUS program, and one representative of DG MARE participated in the survey. The marine projects partaking in this survey predominantly follow a natural scientific, positivist epistemology and apply quantitative-statistical methods. Their research foci range from marine biology and ecology to model development and improvement as well as technological developments. The specific topics included ocean observatory systems, technologies for cleaning marine litter, improvement of stock assessment methods, sustainable fisheries management, new aquaculture technologies, and tipping points of socio-ecological systems. Only two of these projects had consortia that include social scientists. Our survey thus largely covers research fields that did not traditionally incorporate stakeholder engagement methods or the viewpoints of non-academic actors. Most of the surveyed projects started in 2018/2019 and will end in 2021/2022. Although their focus differed, stakeholder engagement was a key tool in all projects. Eighty percent of respondents stated that, in their project, SE was “quite important” or “crucial” for reaching their goals. The main stakeholder groups were fishers, aquaculture farmers, representatives of both industries, policy-makers, environmental management and governmental bodies. Interestingly, 83% of all participants listed research institutions and fellow scientists as a key stakeholder group (Fig. 1). To avoid confusion, we will refer from here on to scientists from within the surveyed projects as “project scientists” or “project members” and will otherwise include scientists from outside the project consortia in the category of “stakeholders”. Additionally, advisory bodies, vaccine and pharmaceutical companies, consumers, fish health specialists, oil and gas companies, renewable energy companies, port authorities, and recreational user groups were listed. The image drawn by these replies is that of a highly diverse stakeholder landscape related to marine science projects, both in terms of societal groups as well as geograhpical focus (see Fig. 2).

To understand what motivated the survey participants to interact with stakeholders, we asked for their main reasons for engaging, giving multiple-choice options based on the categorisation of Stauffacher et al. (2008) (see the “Stakeholder engagement in the marine science” section). The three central motivations to exchange with stakeholders were (1) to identify the research needs of practice actors (63% of respondents), (2) disseminate project results (58%), and (3) establish access to data and information regarding a research problem (54%). These reasons for engagement were followed by co-framing the research process with stakeholders (45%) and implementing new technologies or measures into applied usage (41%). Less than 10% of participants interacted with stakeholders merely to verify their research results or to assist stakeholders in implementing their own research projects. Over half of the respondents are in touch with their stakeholders periodically (i.e. every couple of months), whereas a quarter have weekly or monthly contact and one participant exchanges with stakeholders on a daily basis.

Sixteen out of 17 projects planned to conduct physical meetings such as face-to-face workshops, information events, and face-to-face interviews during their lifespan. Online events had been considered by less than half of the surveyed projects. According to their pre-Covid plans, the year 2020 would have been a “hot phase” of SE for most of the projects. The main engagement activities should have been face-to-face workshops and interviews: both formats that require meeting stakeholders physically and spending time with them.

### Covid-19 impacts on project workflow and outcomes

Although the Covid-19 pandemic has had little impact on the overall goals of the surveyed projects, it has had a clear impact on engagement with stakeholders. In five out of 17 projects, engagement objectives and goals decreased and, in one project, the pandemic increased the overall importance of engagement A quarter of respondents stated that the engagement activities in their project were unaffected by Covid-19. In all other cases, negative impacts were reported on the exchange with practitioners. Except for a few cases where meetings took place under social distancing measures, it is clear that the majority of physical events were either cancelled, delayed or an alternative format was chosen. Online events, on the other hand, were largely carried out as planned (Table 1).

**Table 1:** Overview of engagement formats and stage of implementation under Covid-19 measures (July 2020)

Type of activity	Planned	Implemented	Socially distanced	Delayed	Cancelled	Format changed
Information events	23	2	1	8	6	6
Face-to-face workshops	12	1	-	3	3	5
Conference	11	-	-	7	2	2
Focus groups	3	0	1	1	0	1
Face-to-face interviews	11	2	2	4	1	3
Online interviews	1	1	0	0	0	0
Face-to-face survey	3	0	0	1	1	1
Online survey	3	2	0	1	0	0
Webinar(s)	2	2	0	0	0	0

Source: survey data

With respect to the overall goals of stakeholder engagement, half of the surveyed project scientists believed that the pandemic has had no influence. In one-third of the cases, however, respondents indicated negative impacts to SE. Problems caused by social distancing measures are restrictions of access to events and meetings and the fact that some stakeholder groups are less likely to use online tools than others (see the “Effects on the lives of stakeholders” section). One project placed considerable emphasis on joint writing meetings which had to be cancelled, thus affecting publications planned among project members. Another participant found it easier to reach stakeholders digitally during the lockdown than to assemble them for physical meetings under normal circumstances.

#### Project workflow and deliverables

Six months into the Covid-19 pandemic, over 80% of the survey participants saw the workflow and outcomes of their research project negatively affected. Most commonly (62%), delays in the flow of data to other work packages were anticipated due to the current limitations in engagement possibilities. Twenty percent of respondents even stated that these limitations led to some work packages in their project not being completed at all, or to certain data lacking in work packages. In almost half of the cases, the submission of deliverables (e.g. reports) was or will be delayed whereas a quarter of the participants saw the need for an extension of the project duration. Of the projects which needed to prolong their lifetime, one-third had already negotiated an extension with the EC and two-thirds were aware of how to file for extension but had not yet started the process. Encouragingly, in none of the cases was the overall project objective jeopardised.

### Effects on the lives of stakeholders

We did not directly survey stakeholders, but the survey asked project scientists to give their impressions of how stakeholders are impacted by the pandemic. All respondents described negative impacts of Covid-19 on the circumstances of stakeholders. Their replies to a free text question drew a challenging picture for the marine resources sector at both the local level and international arenas. Demand for fish and seafood markedly decreased during the onset of the pandemic, and the working conditions in aquaculture farms and on fishing vessels were restricted due to the distancing measures. One respondent reported that “fish breeding companies have troubles with access to facilities [and] reduced capacity due to social distancing” (quoted from survey responses). The seafood industry faced heavy losses of revenue due to the closure of hotels, restaurants, catering facilities, canteens in schools and businesses. Participants also highlighted logistical restrictions in transport and border controls for both commodities and workers. Social distancing measures in harbours and onboard of vessels, moreover, created difficulties in the change of marine personnel and crews. In cases where stakeholders from the industry were owners of a business, all these factors—alongside existing pressures such as overfishing and climate change—combined to worsen their economic status and, in the worst instances, financial hardships threatened livelihoods.

Additionally, survey respondents indicated that all stakeholder groups faced common challenges associated with extra family responsibilities due to the need for home schooling and/or extra childcare. On the job market, hiring procedures were on hold and many institutions, be it scientific or governmental, were closed until further notice. One respondent aptly summed up the situation referring to the case of Spain:

*[This] pandemic is a crisis of crises. It has accelerated the vulnerability of more vulnerable sectors of society, and it has made visible the precarious situation of different sectors. In fisheries it has elucidated the weakness of this sector. In Spain [it shows] that we don't have to be so dependent of tourism! […] The pandemic has made visible the problems that already existed before, but now have been accelerated.* (Quoted from survey response)

#### A changed relationship with stakeholders?

**Fig. 3:**
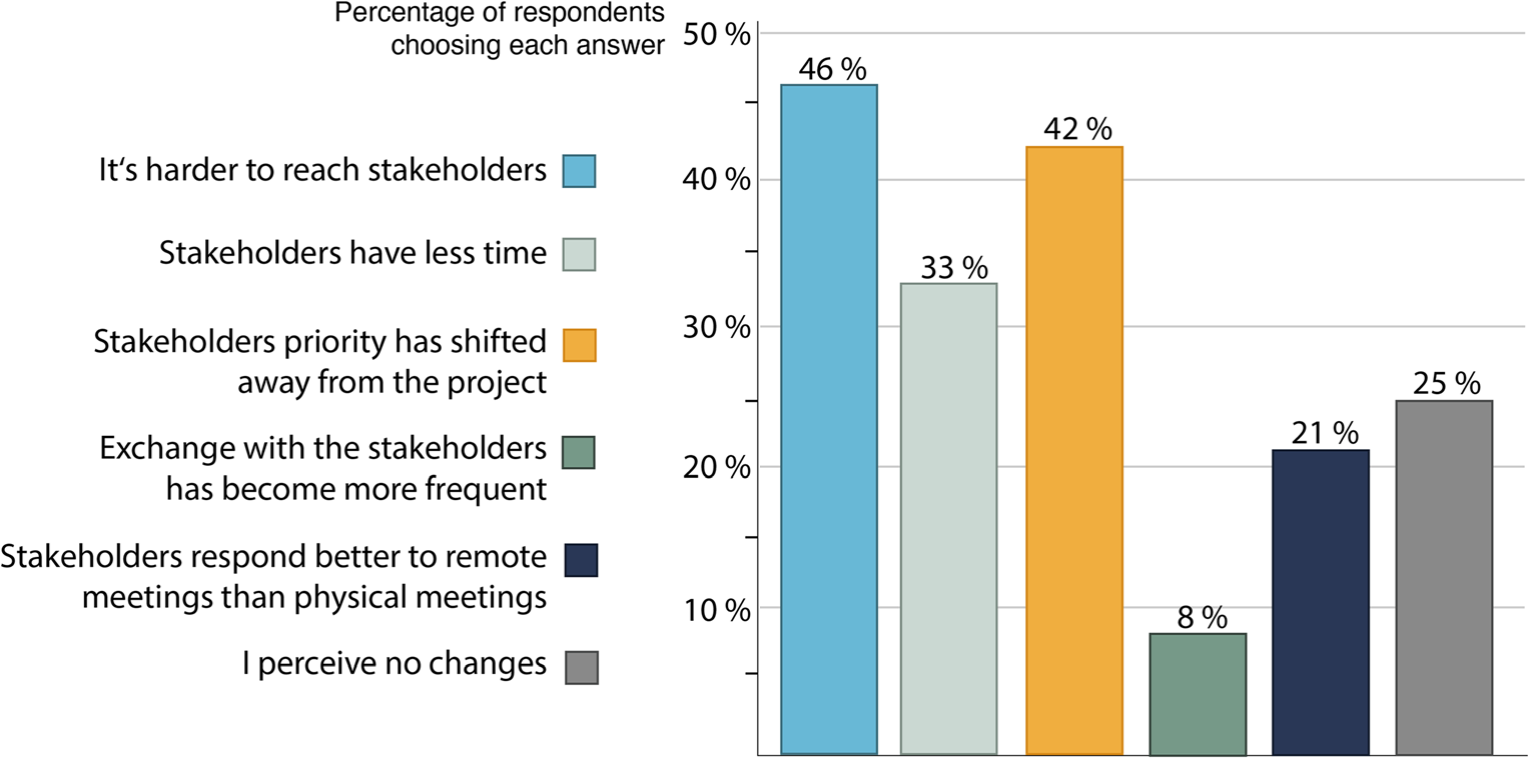
Changes in the relationship with stakeholders (multiple responses possible). Source: survey data

The overall relationship between project scientists and stakeholders was largely perceived to be unchanged; only 20% of respondents stated that their connection with practice partners had worsened. Looking at the details of this relationship, however, a more complicated picture arises. For 45% of respondents, the social distancing measures made it harder to reach stakeholders, and 41% perceived that stakeholders’ priorities have shifted away from the research project. Moreover, one-third of participants stated that stakeholders appear to have less time for meetings, be they virtual or physical, than before the start of lockdowns and distancing (Fig. 3).

Although it “has become more difficult to plan upcoming meetings, plans, or any other activity”, the distancing measures were perceived to “bring momentum with virtual meetings” and make it “possible to host more meetings that would not originally take place” offline (quoted from survey response). Twenty percent of participants indicated that the shift to online methods of interaction made it easier to reach stakeholders and, in only 2% of the cases, the contact with stakeholders even became more frequent than before the pandemic (Fig. 3).

Although it was perceived to be easier to meet with stakeholders online, one-third of respondents were unable to use online methods to contact at least one of their stakeholder groups. Respondents reported that artisanal fishers and aquaculture farmers were often not accustomed to working online and/or had limited internet access. Besides these technical issues, it was observed that “fishers’ priorities have moved away from scientific research to address more important concerns such as reduced markets and additional costs related to increased security and hygiene measures” (quoted from survey response). Furthermore, due to heightened workloads or difficulties in (re)arranging their person lives, certain groups of business people and policy-makers were less responsive to email.

### Social from a distance—Engaging stakeholders during the pandemic

Since regular physical meetings were not possible during this survey period, and may not be possible in the foreseeable future, many research projects are currently developing alternative solutions to engage with their stakeholders. How are the project members responsible for engagement coping with the social distancing measures, and which alternatives do they apply?

#### Alternative engagement formats

A variety of activities were planned, and due to Covid-19, alternative formats were chosen (Table 2). The most common replacement was the implementation of webinars instead of physical information events or face-to-face workshops. In two cases, such events were replaced by online workshops. Other substitutions were the use of emails to circulate information among stakeholders and the creation of posters and infographics shared with fishing associations and broader audiences online. Two project scientists stated that they replaced a stakeholder conference with a webinar or an online workshop. Online interviews were chosen in several cases to substitute information events, face-to-face workshops, and face-to-face interviews. In another case, such interviews were replaced with an online survey or conversations via WhatsApp.

**Table 2:** Alternative engagement methods in cases where the format was changed. Note, cancelled or delayed activities are not included.

Type of activity	Planned	Webinar	Online workshop	Online conference	Online focus groups	Online interviews	Online survey	Other
Information events	23	4	2	-	-	1	1	2^1^
Face-to-face workshops	12	4	2	-	-	1	-	-
Conference	11	2	1	-	-	-	-	-
Focus groups	3	-	-	-	1	-	-	-
Face-to-face interviews	11	-	-	-	1	1	-	2^2^
Face-to-face survey	3	-	-	-	-	-	1	-
Online interviews	1	-	-	-	-	-	-	-
Online survey	3	-	-	-	-	-	-	-
Webinar(s)	2	-	-	-	-	-	-	-

^1^(1) Emails; (2) posters, infographics through media and to post at the fishers’ associations

^2^(1) Not decided yet; (2) WhatsApp, phone

Source: survey data

**Fig. 4:**
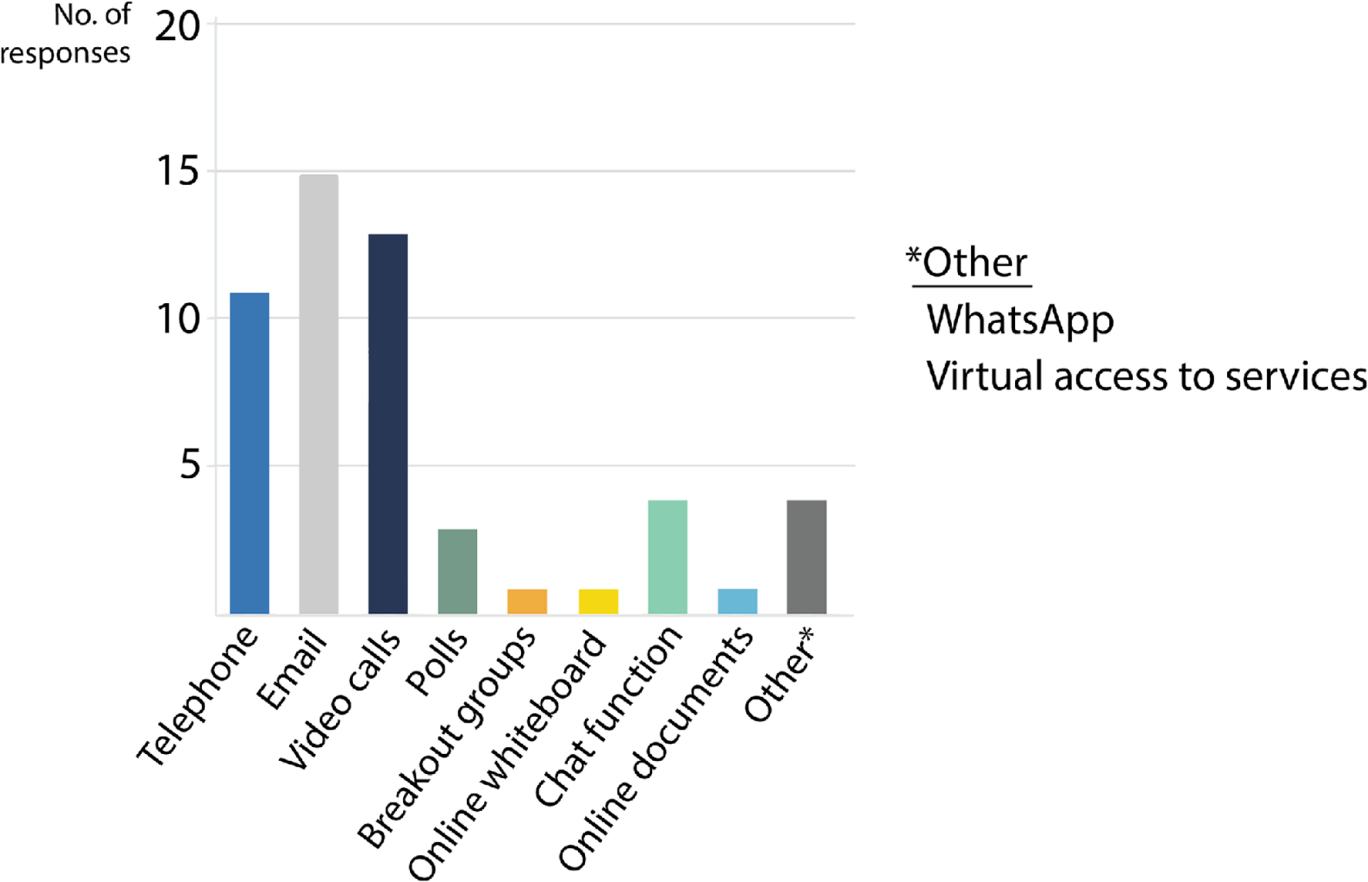
Methods chosen for online engagement with stakeholders. Source: own figure based on survey data

Generally, the array of methods for online engagement was fairly broad, but certain formats were clearly preferred. The most common methods were the “classic” ones such as telephone calls and emails complemented by video calls. Online polls, chat programs, and breakout groups or online documents were seldomly used by the project members to communicate with their stakeholders during the pandemic (Fig. 4). One respondent stressed the importance of WhatsApp for keeping in touch with stakeholders, especially with groups that were difficult to reach via email or video calls.

#### Accounting for Covid-19 in engagement outcomes

Respondents reported that Covid-19 changed the lives of their stakeholders in different ways (see the “Effects on the lives of stakeholders” section). As argued by Fell et al. (2020), research results derived from current engagement activities will be highly influenced by the pandemic. We, therefore, asked survey participants if they implemented any specific measures to help ensure that their results were also valid after the pandemic. Of the measures proposed by Fell et al. (2020) (see the “Academic life in a new, digital mode” section), one-third of the respondents stated that they collected additional contextual information about Covid-19 implications on the marine sector, e.g. data on the impact on fishing effort and catch quantified in fisheries statistics. Similarly, a third of survey participants included questions on self-reported behavioural changes by their stakeholders such as changes in fishing routines or the generation of alternative income. Another third of the respondents addressed the stakeholders’ experiences of the crisis on the individual or professional level when talking with them and considered this when interpreting their results. A lower proportion of participants collected additional demographic data and potential changes in, for example, the employment situation of stakeholders (16%) or directly addressed the impact of social distancing measures on the cooperation between project members and stakeholders (8%). Furthermore, 45% of the participants stated that no measures were taken to help ensure that data and results obtained from SE during the pandemic will be comparable to those obtained pre- and post-pandemic.

#### Evaluation of alternative engagement formats

**Fig. 5:**
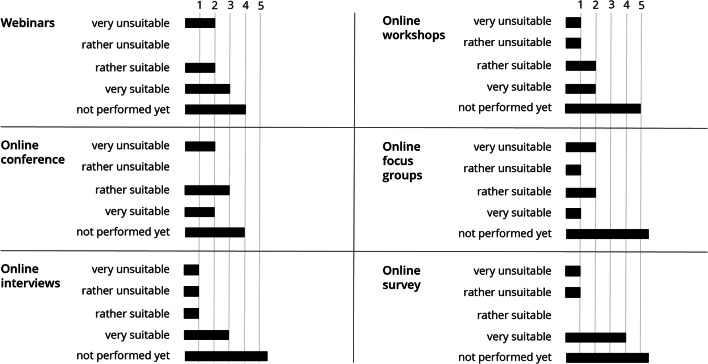
Assessment of alternative, socially distanced engagement methods. Numbers represent replies per answer option. Source: own figure based on survey data

Many engagement activities were cancelled or postponed due to social distancing measures (Table 1). The most common substitute formats were webinars and online workshops as well as the use of emails, telephone, and video calls. Although many alternative methods had not yet been applied in late July 2020 (Fig. 5), among methods that had been used, webinars, online workshops, and online conferences were rated more positively than negatively. Online focus groups, on the other hand, received both positive and negative evaluations. Online surveys and interviews had the most positive responses, and the latter was viewed as a suitable tool for replacing physical meetings with stakeholders (Fig. 5).

Online tools as a general substitute for face-to-face contacts were perceived to be “very suitable for people with relevant background and skills (for example oil rig operators, port authority executives, etc.)”, but the use of technology should be kept as easy as possible to not demand too much time or effort from the stakeholders (quoted from survey data). Only one survey respondent stressed that focus groups with many participants require good preparation and coordination. Digital meetings were viewed as less productive than face-to-face meetings and provide a lower level of information. Another point raised was that, even though stakeholders might have access to online tools and technologies, they might not be accustomed to frequently attending lengthy video meetings.

There was a broad willingness to continue working with digital meeting formats also after social distancing measures are lifted. Webinars and e-conferences were viewed as useful tools especially in international projects. Five respondents stated that they have been using tools such as WhatsApp, Skype, and webinars in their projects before and will continue to do so after the pandemic. In two cases, digital technologies will be used for a while after the end of lockdowns to keep project members and stakeholders safe. There were, however, also two strong “No’s” on the continued use of online engagement measures, highlighting the importance of face-to-face meetings as key communication tools with stakeholders.

## Discussion and recommendations for practice

### Engagement is challenged, but ongoing

Forty percent of current EU H2020 marine science projects provided feedback, as well as several non-H2020 projects. Although the majority of these projects could be considered natural science, all projects are engaging with stakeholders due to either intrinsic motivation and/or in response to changing H2020 funding requirements (see the “Increased importance of SE in research funding” section; cf. Mackinson and Holm 2020). Although projects have maintained their goals, engagement has shifted to online technologies. Due to changes in daily routines and challenges to both project scientists and stakeholders (cf. “Academic life in a new, digital mode”, “Social distancing and SE in EU projects”, and “Covid-19 impacts on fishing and the marine sector”), it is not surprising that the priority of engagement activities has decreased.

There were several motivations for engaging stakeholders including the identification of research needs outside of the academic world, access to data and information, and disseminating project results (cf. “Overview of surveyed projects, motivations, and methods of engagement”). On the scale of engagement levels distinguished by Stauffacher et al. (2008) from low (inform stakeholders about a project) to high (conducting cooperative projects to address a research question posed by stakeholders), both ends of this “engagement spectrum” are under-represented in our survey responses. This suggested that the engagement activities in these (EU) marine science projects go beyond unidirectional information events, but that they also do not fully embrace an approach of knowledge co-creation with stakeholder.

The survey indicated that 2020 was envisioned to be engagement-intensive for most projects. The Covid-19 pandemic clearly reduced activities and caused a shift to unforeseen methods. Only in three cases did projects originally plan online methods and their engagement methods were largely unchanged. Most physical meetings were either cancelled, delayed or their format was changed to online tools. In terms of the workflow of projects, the pandemic primarily caused delays in the submission of deliverables and in the data flow between work packages. A direct effect of these delays is the need for extending the project duration, an issue faced by a quarter of the surveyed projects. As such delays were foreseen by the EC in time, a good procedure for project extensions is already in place ensuring that ongoing research can be completed (cf. EC 2020b).

#### Negative impacts outweigh positive ones

The survey respondents observed both positive and negative impacts of the pandemic, with the latter clearly outweighing the former. The focus of observation lies on the effects on the fishing and aquaculture sector, and only few participants outline how other stakeholder groups are affected. These responses mirror statements on the effects of Covid-19 on the marine sector in recently published, peer-reviewed literature (see the “Covid-19 impacts on fishing and the marine sector” section). On the upside, some stakeholders are reported to respond better to digital meetings than physical ones as travel time is reduced and it is less effort to meet online (cf. “Academic life in a new, digital mode”; Klöwer et al. 2020). On the downside, however, the main problems caused by the distancing rules are as follows:

- It being harder to reach stakeholders digitally
- Stakeholders’ priorities shifting away from participating in research projects
- Stakeholders having less time to spend on participation

Besides the impacts listed in our multiple-choice questions, additional negative effects of social distancing were brought up by the survey respondents. One project, for instance, struggles with the cancellation of their project-internal face-to-face writing meetings and joint publications. The pandemic therefore not only poses challenges to SE but also to project-internal workflows. All in all, the Covid-19 pandemic makes SE more difficult in most cases (69%), whereas only 16% of respondents see positive effects and 14% perceive no changes at all.

Most projects covered by our survey were in the mid of their lifetime, and engagement efforts should have peaked this year. The fact that numerous activities were either cancelled, delayed or replaced shows that engagement in research projects is severely disrupted, and the magnitude of the impacts of this disruption will likely not be apparent until later stages in the projects. In practice, this means that amendments, forms, and applications for changes in the work program will be needed. The increased administrative work for project scientists may decrease the time available for engagement later in the projects.

#### A shift towards exclusion?

Webinars and online workshops have become the preferred choices for SE in times of Covid-19. In terms of the tools used, “classical” methods like video meetings outweigh technologies like online whiteboards or shared online documents. This choice is easily explained by the fact that most scientists are likely already familiar with programs such as Skype and Zoom, but not so much with more interactive software such as MURAL (MURAL 2020) that is currently rapidly developing. Looking into the future, only 8% of the respondents stated that they will return completely to the methods used pre-Covid, while all other participants favoured extending their use of online tools and digital meetings. This tendency agrees with the current trend of shifting more and more work processes to the digital world, even though it bears the danger of excluding certain stakeholders who have limited access to online technologies or are not accustomed to using them (cf. Beaunoyer et al. 2020). This concern is mirrored by our survey which showed that 30% of participants could not reach at least one of their stakeholder groups via online tools, and this survey was focused on the European region and not projects conducted in developing countries where larger challenges are envisioned. There is a danger of exclusion (Beaunoyer et al. 2020) that perpetuates the imbalance of power and decision-making capacities, and different ways of knowing (Colvin et al. 2020) that participatory science attempts to bring together. The ways in which we identify stakeholders and decide who is allowed to have a say or, in other words, our “definition of legitimate ‘stakeholderness’” (Linke and Jentoft 2016: 6; Metzger et al. 2017) is a first hurdle to overcome when using a participatory approach. The digitalisation brought about by the Covid-19 pandemic represents a new hurdle. The main question may have shifted from “Who does not want to participate in our research project?” to “Who is not technically able to participate?”. Regarding digital engagement of stakeholders in marine science projects, small-scale fishers and aquaculture farmers (groups that are often at the margins of engagement activities) are likely to be poorly represented. A “relocation” of all meetings to digital formats can, thus, exacerbate “technology-related societal inequalities” (Beaunoyer et al. 2020). We, as initiators of engagement activities, must be aware of this fact—especially in projects aiming to solve complex environmental problems that rely on cooperation with various societal actors (cf. “A growing interest in exchange with practitioners”; Irwin and Horst 2016).

As we conducted our survey early in the pandemic and as most surveyed projects continue for at least another year or two, it is likely too soon to comment on the impact of the Covid-19 pandemic on the overall success of these projects. The survey results, however, demonstrate clear delays in data collection, project work flow, and the submission of deliverables. Fortunately, the EC has prepared for extensions if required. The fact that 45% of respondents indicated that no steps were taken to help ensure that data and results obtained during the pandemic were comparable to those collected pre- (and eventually post-) pandemic is concerning. The conclusions of projects attempting to account for the potential influence of changes in engagement methods and the status of stakeholders on SE will be particularly interesting and useful.

### Recommendations for “distanced engagement”

In the wake of a global pandemic, the challenging task of engaging stakeholders in research projects has become ever more demanding. With awareness of the problems we are facing, however, we can still successfully co-create the data for our research together with practice partners, even in times of social distancing. Based on the results of our survey and our personal experiences with engaging stakeholders during the pandemic, we provide a number of suggestions for engaging with stakeholders when physical meetings are not an option. These suggestions may also be important in the decisions made by projects engaging stakeholders in a future (post-pandemic) world in which digital meeting formats will likely play a larger and larger role.

1. Know your stakeholders (better than before).
  When hosting online meetings, be aware of the different stakeholder groups and their access to online tools (cf. “Engagement is challenged, but ongoing”; Beaunoyer et al. 2020). Simultaneously, it is important to develop an awareness of the problems that different groups and sectors are facing due to the pandemic, how their circumstances change, and how this affects their willingness and ability to engage. We can then deduct which formats of engagement are suitable for different groups and how we can keep the exchange alive.
2. Strengthen existing relationships.
  Everyone has less time during lock-downs because of added responsibilities. Lengthy online meetings might exceed our stakeholders’ capacities. Work toward fulfilling your project’s goals with different forms of engagement; e.g. consider briefer one-on-one conversations. Personal exchange, even if informal, strengthens existing relationships. Choosing means of communication stakeholders respond to well and reassuring them that you are aware of the hardships they face due to the pandemic intensifies ongoing and new relationships.
3. Do not go 100% digital.
  Beaunoyer et al. (2020) underlined how vital it is to be aware of the stakeholder groups that are hard to reach digitally, and to develop alternative ways of contact with those groups to avoid a culture of exclusion. Whereas it is the task of government bodies to provide quality digital infrastructure, it is our responsibility to diversify the ways in which we communicate. One should consider not only writing emails and holding video meetings but also using the telephone, messaging apps, or even retreating to “old school” forms of communication such as posters on notice boards or sector-specific newspapers.

4) Re-think your offline methods.
  Meetings with fewer participants tend to be more productive than large group calls. Long video meetings are tiring for everyone, especially for stakeholders who are not accustomed to using online tools (cf. “Academic life in a new, digital mode”; survey results; Beaunoyer et al. 2020). Moreover, online meetings, even if intended to have a similar structure as offline, require different and more thorough preparation. As hosts of such meetings, we should be well-versed with the technology we use, able to quickly solve technical issues, and able to moderate meetings differently than we would offline.
5) Stay flexible and keep it simple.
  The engagement strategies planned at the start of a project often no longer apply during the Covid-19 pandemic. Strategies need to be revisited to elaborate which research questions need to be answered in dialogue with stakeholders and what timeline was foreseen. This project introspection is needed to redesign the engagement strategy, include alternative methods, and develop a new project timeline. While online meetings provide a great opportunity for exchanging over long distances and keeping CO_2_ emissions low, they may be inappropriate if some stakeholders/groups are poorly versed in virtual tools. For particular groups, “simple” communication means such as WhatsApp and phone calls might be a lot more useful for maintaining the conversation.
6) Learn lessons for post-pandemic engagement.
  When deciding which new methods to maintain after the pandemic, consider which stakeholder groups’ voices are strengthened or weakened in your project. The decision to go more digital provides advantages, but bears the danger of restricting who has and does not have access to your project. There is an inherent potential for bias.
7) Account for the Covid-19 circumstances in your research results.
  Fell et al. (2020) stressed the importance of accounting for the circumstances of the pandemic in the interpretation of research results obtained during the pandemic, and we echo this sentiment. Collecting additional information on the challenges posed by the pandemic to different stakeholder groups (e.g. in different regions), be it quantitative or qualitative information on the hardships faced by stakeholders, will be important. Including this information can be as simple as accompanying workshop reports and co-developed research results with a note outlining the specific circumstances in a specific location or a more general description of how the pandemic affected the sectors of fisheries, aquaculture, and marine resource management in any given region.

## Conclusions

The process of stakeholder engagement is an especially communicative, social activity that relies on close relationships and face-to-face meetings and is, therefore, severely affected by the social distancing measures imposed to stop the spread of Covid-19. Digital engagement has become the new norm and can offer a greater continuity of exchange at reduced effort and with a much lower carbon footprint. An important disadvantage, however, is the potential to exclude certain societal groups. An increased awareness is needed of who our stakeholders are, how they are affected by the pandemic, and how we can effectively engage them during social distancing. A follow-up survey similar to the one presented here is needed after the pandemic to assess the magnitude of impacts of Covid-19 on project results and to document best practice emerging as more experience has been gained. In this future research, it is critical to gain the perceptions of stakeholders on science engagement as only scientists were surveyed in the present study. Our recommendations constitute a check list that can be used by project scientists, especially those not yet familiar with suitable methods for stakeholder engagement, to critique their engagement practices before, during, and after the Covid-19 pandemic. We hope that this study contributes to finding improved ways of exchanging with a wide range of practice partners within and outside European marine science to increase the societal relevance and impact of science.
